# Retention and mortality on antiretroviral therapy in sub‐Saharan Africa: collaborative analyses of HIV treatment programmes

**DOI:** 10.1002/jia2.25084

**Published:** 2018-02-26

**Authors:** Andreas D Haas, Elizabeth Zaniewski, Nanina Anderegg, Nathan Ford, Matthew P Fox, Michael Vinikoor, François Dabis, Denis Nash, Jean d'Amour Sinayobye, Thêodore Niyongabo, Aristophane Tanon, Armel Poda, Adebola A Adedimeji, Andrew Edmonds, Mary‐Ann Davies, Matthias Egger, the African regions of the International epidemiologic Databases to Evaluate AIDS (IeDEA)

**Affiliations:** ^1^ Institute of Social & Preventive Medicine University of Bern Bern Switzerland; ^2^ World Health Organisation Geneva Switzerland; ^3^ Health Economics and Epidemiology Research Office Department of Internal Medicine School of Clinical Medicine Faculty of Health Sciences University of the Witwatersrand Johannesburg South Africa; ^4^ Department of Epidemiology Boston University School of Public Health Boston MA USA; ^5^ Department of Global Health Boston University School of Public Health Boston MA USA; ^6^ Department of Medicine University of Alabama at Birmingham Birmingham AL USA; ^7^ Centre for Infectious Disease Research in Zambia Lusaka Zambia; ^8^ School of Medicine University of Zambia Lusaka Zambia; ^9^ ISPED Centre Inserm U1219‐Bordeaux Population Health Université de Bordeaux Bordeaux France; ^10^ Department of Epidemiology and Biostatistics City University of New York, School of Public Health New York NY USA; ^11^ Institute for Implementation Science in Population Health City University of New York New York NY USA; ^12^ Rwanda Military Hospital Kigali Rwanda; ^13^ Centre National de Reference en Matiere de VIH/SIDA (CNR) Bujumbura Burundi; ^14^ Service de Maladies Infectieuses et Tropicales (SMIT) CHU de Treichville Abidjan Cote d'Ivoire; ^15^ Institut Supérieur des Sciences de la santé Université Polytechnique de Bobo‐Dioulasso Bobo‐Dioulasso Burkina Faso; ^16^ Department of Epidemiology and Population Health Albert Einstein College of Medicine and Montefiore Medical Center Bronx NY USA; ^17^ Department of Epidemiology The University of North Carolina at Chapel Hill Chapel Hill NC USA; ^18^ Centre for Infectious Disease Epidemiology and Research University of Cape Town Cape Town South Africa

**Keywords:** retention, mortality, loss to follow‐up, antiretroviral therapy, sub‐Saharan Africa

## Abstract

**Introduction:**

By 2020, 90% of all people diagnosed with HIV should receive long‐term combination antiretroviral therapy (ART). In sub‐Saharan Africa, this target is threatened by loss to follow‐up in ART programmes. The proportion of people retained on ART long‐term cannot be easily determined, because individuals classified as lost to follow‐up, may have self‐transferred to another HIV treatment programme, or may have died. We describe retention on ART in sub‐Saharan Africa, first based on observed data as recorded in the clinic databases, and second adjusted for undocumented deaths and self‐transfers.

**Methods:**

We analysed data from HIV‐infected adults and children initiating ART between 2009 and 2014 at a sub‐Saharan African HIV treatment programme participating in the International epidemiology Databases to Evaluate AIDS (IeDEA). We used the Kaplan–Meier method to calculate the cumulative incidence of retention on ART and the Aalen–Johansen method to calculate the cumulative incidences of death, loss to follow‐up, and stopping ART. We used inverse probability weighting to adjust clinic data for undocumented mortality and self‐transfer, based on estimates from a recent systematic review and meta‐analysis.

**Results:**

We included 505,634 patients: 12,848 (2.5%) from Central Africa, 109,233 (21.6%) from East Africa, 347,343 (68.7%) from Southern Africa and 36,210 (7.2%) from West Africa. In crude analyses of observed clinic data, 52.1% of patients were retained on ART, 41.8% were lost to follow‐up and 6.0% had died 5 years after ART initiation. After accounting for undocumented deaths and self‐transfers, we estimated that 66.6% of patients were retained on ART, 18.8% had stopped ART and 14.7% had died at 5 years.

**Conclusions:**

Improving long‐term retention on ART will be crucial to attaining the 90% on ART target. Naïve analyses of HIV cohort studies, which do not account for undocumented mortality and self‐transfer of patients, may severely underestimate both mortality and retention on ART.

## 1 Introduction

Over the past 15 years antiretroviral therapy (ART) has been scaled up massively in low‐ and middle‐income countries: by mid‐2017 globally almost 21 million people were receiving ART 1. According to modelling by the Joint United Nations Programme on HIV/AIDS (UNAIDS), 1.2 million deaths have been averted due to ART and 35% of the worldwide new HIV infections prevented by ART 1. In 2014, UNAIDS launched the “90‐90‐90” targets and “treatment as prevention” became a priority of the global response to the HIV epidemic 2, 3, 4, 5, 6: by 2020, 90% of all people living with HIV know their status, 90% of those diagnosed receive ART, and 90% of the people on ART have undetectable viral load. The 90‐90‐90 strategy aims to ensure that the majority of people living with HIV are on successful ART and no longer transmit the virus, thus ending the AIDS epidemic as a public health threat by 2030.

In line with the 90‐90‐90 strategy, the latest HIV treatment guidelines recommend that all people living with HIV should initiate ART regardless of clinical stage and CD4 cell count. A key barrier to ART uptake, the requirement to undergo CD4 testing prior to ART initiation, was thus removed 4, 7, facilitating early initiation of ART. However, persons successfully initiated on ART need to continue ART for life. Patients who stop taking ART rapidly lose the benefits of viral suppression and may transmit the virus or fall sick 8. Retaining people on ART is a challenge in sub‐Saharan Africa. A recent systematic review of cohort studies from sub‐Saharan Africa showed that every third ART patient was classified as lost to follow‐up within 3 years of starting ART 9. The proportion of patients who stopped taking ART cannot be easily determined from clinic data, because patients classified as lost to follow‐up may have self‐transferred to another HIV treatment programme, or may have died 10, 11, 12.

The true status of persons lost to follow‐up can be determined through tracing by phone calls and home visits. A recent meta‐analysis of tracing studies from African HIV programmes showed that about one‐third of all patients lost to follow‐up had self‐transferred to another ART facility, one‐third had stopped taking ART, and one‐third had died 11. Tracing outcomes can be used to adjust estimates for mortality and treatment discontinuation from cohort studies 13, 14, 15, 16.

We first describe mortality, loss to follow‐up and retention on ART among HIV‐infected children and adults who initiated ART in sub‐Saharan Africa, as recorded by the HIV care programmes. We then adjust analyses for undocumented mortality and self‐transfer to estimate the proportion of patients who died, stopped ART, and are retained on ART in Central, East, West and Southern Africa.

## 2 Methods

### 2.1 Data sources

The International epidemiology Databases to Evaluate AIDS (IeDEA) is a global consortium of HIV cohort studies. We included all HIV treatment cohorts that participate in IeDEA Central Africa, East Africa, Southern Africa, and West Africa 17. Fifty‐seven cohorts from 22 countries contributed data, including 3 cohorts from Burundi, 2 from the Democratic Republic of the Congo, 10 from Rwanda (Central African region); 2 cohorts from Kenya, 1 from Tanzania, 4 from Uganda (East Africa); 1 cohort from Lesotho, 2 from Malawi, 1 from Mozambique, 7 from South Africa, 1 from Zambia and 2 from Zimbabwe (Southern Africa); 1 cohort from Benin, 2 from Burkina Faso, 8 from Côte d'Ivoire, 1 from Ghana, 1 from Guinea, 1 from Guinea‐Bissau, 2 from Mali, 2 from Nigeria, 2 from Senegal and 1 from Togo (West Africa). In Central Africa and West Africa most participating sites were urban regional, provincial, or university hospitals. In East and Southern Africa they were health centres, district hospitals and regional, provincial, and university hospital from urban and rural settings. The number of patients ranged from 42 patients in the Nyarugunga clinic in Rwanda, to almost 230,000 patients at the Centre for Infectious Disease Research in Zambia (CIDRZ). Data are collected during routine care at ART initiation and at each follow‐up visit, including socio‐demographic information, date of ART start, type of ART, and (where available) CD4 counts and viral load measurements. Regional IeDEA data centres sent de‐identified data to the University of Bern, Switzerland for cleaning and analysis.

### 2.2 Eligibility criteria

We included HIV‐infected adults and children who initiated ART during 2009 to 2014 at one of the HIV treatment programmes that participate in IeDEA Central Africa, East Africa, Southern Africa and West Africa. We excluded patients known not to be ART‐naïve at enrolment. Individuals who received antiretroviral drugs to prevent mother‐to‐child transmission or for pre‐ or post‐exposure prophylaxis prior to starting ART were considered ART experienced and excluded.

### 2.3 Outcomes

For the analyses of the data as recorded in the clinical databases, outcomes were retention on ART, loss to follow‐up, and death. Outcomes were mutually exclusive. For the definition of loss to follow‐up, we calculated a patient's next appointment date as the last visit date plus a patient's most recent gap between two consecutive visits that was in accordance with the visit schedule of the clinic. We then classified patients more than 90 days late for their next clinic appointment who did not return to care during the study period as lost to follow‐up on the day of the first missed appointment. In the adjusted analyses, outcomes were retention on ART, death, and stopping ART. We assumed that patients recorded as lost to follow‐up by a treatment programme who were not classified as self‐transfer or unrecorded death were alive and had stopped ART.

### 2.4 Statistical analyses

We used descriptive statistics to describe patient's characteristics at initiation of ART, stratified by IeDEA region. We used survival analysis methods to analyse time to loss to follow‐up, stopping ART and death. We followed patients from ART initiation for a maximum of 5 years, censoring follow‐up time of patients who transferred to another clinic or programme that was not covered by IeDEA on the date of transfer. Individuals retained on ART were censored 90 days before database closure, when they ceased to be at risk of loss to follow‐up. We used the Kaplan–Meier estimator to calculate the cumulative probability of retention on ART and the Aalen‐Johansen estimator to calculate the cumulative incidences of documented death or loss to follow‐up and stopping ART. Death and loss to follow‐up or stopping ART were competing risks 18.

In a second, adjusted analysis, we adjusted estimates to account for undocumented mortality and self‐transfer of patients to another HIV treatment programme using inverse probability weighting. Weights were based on the results from a recent meta‐analysis of 32 studies of 20,365 patients who were lost to follow‐up on ART in sub‐Saharan Africa 11. In these studies, patients were traced by phone calls or home visits. In South Africa, their vital status was assessed through the vital registration system 11. This meta‐analysis showed that mortality in patients lost to follow‐up declined in recent years, whereas undocumented transfers to other clinics increased 11. Based on these data, we assumed that 20.8% of all patients recorded as loss to follow‐up had died, and 35.9% had self‐transferred to another facility. These estimates correspond to the meta‐analysis' estimates for the year 2012, which was the mid‐study year of our analysis. Patients classified as loss to follow‐up were down weighted by 1 minus the probability that they had been misclassified due to underreporting of death or transfer. Patients classified as dead or retained in care were up weighted by the inverse probability of observing death or retention, given underreporting of death and transfer. The formulas used to calculate the weights are given in Box 1. In a sensitivity analysis, we calculated weights based on the upper and lower limits of the 95% confidence intervals (CIs) around the estimates for mortality (11.3 to 35.1%) and self‐transfer (16.8 to 60.9%) among patients lost to follow‐up 11.

Box 1 **Calculation of weights to adjust for undocumented mortality and undocumented transfer**

We calculated the weights using the following formulas:

W_LTF _= 1 − p_dead_ − p_transfer_, for patients lost to follow‐up (LTF)
  $$W_{\mathrm{dead}}=\frac{1}{\frac{n_{\mathrm{dead}}}{n_{\mathrm{dead}}+p_{\mathrm{dead}}*n_{\mathrm{LTF}}}},\;\mathrm{for}\;\mathrm{patients}\;\mathrm{who}\;\mathrm{died}$$
  $$W_{\mathrm{RIC}}=\frac{1}{\frac{n_{\mathrm{RIC}}}{n_{\mathrm{RIC}}+p_{\mathrm{transfer}}*n_{\mathrm{LTF}}}},\;\mathrm{for}\;\mathrm{patients}\;\mathrm{retained}\;\mathrm{in}\;\mathrm{care}\;(\mathrm{RIC})$$

where

n_dead _= number of patients known to be dead
n_LTF _= number of patients lost to follow‐up (LTF)
n_RIC _= number of patients retained in care
p_dead_ = proportion of patients LTF who were found to have died (derived from 11)
p_transfer _= proportion of patients LTF who silently transferred (derived from 11)

### 2.5 Ethical considerations

Local review boards and ethics committees approved the use of the data included in this study. The Cantonal Ethics Committee of the Canton of Bern, Switzerland, approved data merging and the collaborative analyses. Local review boards and the Cantonal Ethics Committee of the Canton of Bern waived the requirement to obtain informed consent.

## 3 Results and Discussion

We included 505,634 patients: 12,848 (2.5%) from Central Africa, 109,233 (21.6%) from East Africa, 347,343 (68.7%) from Southern Africa and 36,210 (7.2%) from West Africa. The median (interquartile range, IQR) age at ART initiation was 33.7 years (27.1 to 41.0); 22,939 (4.5%) patients were under 5 years, 21,115 (4.2%) were aged 5 to 14 years and 461,580 (91.3%) were 15 years or older. The majority (318,491; 63.0%) were female. Median CD4 cell count (IQR) at ART initiation was 200 cells/μL (102 to 311 cells/μL). Patient characteristics are shown in Table 1.

**Table 1:** Characteristics of patients at the start of antiretroviral therapy by IeDEA region

	IeDEA region				
	Central Africa 12,848 (2.5%)	East Africa 109,233 (21.6%)	Southern Africa 347,343 (68.7%)	West Africa 36,210 (7.2%)	Total 505,634 (100.0%)
Age					
<5	515 (4.0%)	8824 (8.1%)	12,317 (3.5%)	1283 (3.5%)	22,939 (4.5%)
5 to 14	866 (6.7%)	5145 (4.7%)	13,733 (4.0%)	1371 (3.8%)	21,115 (4.2%)
15+	11,467 (89.3%)	95,264 (87.2%)	321,293 (92.5%)	33,556 (92.7%)	461,580 (91.3%)
Median (IQR)	33.1 (25.8 to 41.2)	32.8 (25.2 to 41.0)	33.8 (27.5 to 40.7)	35.7 (29.0 to 43.6)	33.7 (27.1 to 41.0)
Sex					
Male	4504 (35.1%)	37,929 (34.7%)	132,159 (38.0%)	12,551 (34.7%)	187,143 (37.0%)
Female	8344 (64.9%)	71,304 (65.3%)	215,184 (62.0%)	23,659 (65.3%)	318,491 (63.0%)
Year of ART initiation					
2009 to 2010	5012 (39.0%)	38,160 (34.9%)	107,392 (30.9%)	16,044 (44.3%)	166,608 (33.0%)
2011 to 2012	4521 (35.2%)	43,779 (40.1%)	114,656 (33.0%)	12,239 (33.8%)	175,195 (34.6%)
2013 to 2014	3315 (25.8%)	27,294 (25.0%)	125,295 (36.1%)	7927 (21.9%)	163,831 (32.4%)
CD4 cell count (cells/μL)					
<200	2632 (30.6%)	34,101 (51.5%)	109,256 (50.2%)	11,710 (50.7%)	157,699 (50.0%)
200 to 349	4073 (47.4%)	19,882 (30.1%)	72,845 (33.5%)	6288 (27.2%)	103,088 (32.7%)
350 to 500	999 (11.6%)	5675 (8.6%)	18,837 (8.7%)	2293 (9.9%)	27,804 (8.8%)
>500	892 (10.4%)	6495 (9.8%)	16,809 (7.7%)	2797 (12.1%)	26,993 (8.6%)
Median (IQR)	276 (172 to 343)	194 (87 to 312)	199 (106 to 307)	196 (83 to 329)	200 (102 to 311)
Missing	4252 (33.1%)	43,080 (39.4%)	12,9596 (37.3%)	131,22 (36.2%)	190,050 (37.6%)
WHO clinical stage					
I	2752 (36.1%)	28,484 (32.2%)	101,338 (37.8%)	6084 (33.5%)	138,658 (36.3%)
II	1776 (23.3%)	27,074 (30.6%)	56,979 (21.3%)	3557 (19.6%)	89,386 (23.4%)
III	2579 (33.8%)	25,622 (29.0%)	95,842 (35.8%)	6675 (36.7%)	130,718 (34.2%)
IV	519 (6.8%)	7249 (8.2%)	13,914 (5.2%)	1865 (10.3%)	23,547 (6.2%)
Missing	5222 (40.6%)	20,804 (19.0%)	79,270 (22.8%)	18,029 (49.8%)	123,325 (24.4%)

Data are number (%) of patients if not otherwise stated. ART, antiretroviral therapy; IeDEA, International epidemiology Databases to Evaluate AIDS. WHO, World Health Organization. CD4 cell counts and WHO clinical stage were assessed at ART initiation.

Data are stratified by IeDEA region. Central Africa: Burundi, Dem. Rep. of Congo and Rwanda; East Africa: Kenya, Tanzania, Uganda; Southern Africa: Lesotho, Malawi, Mozambique, South Africa, Zambia and Zimbabwe; West Africa: Benin, Burkina Faso, Côte d'Ivoire, Ghana, Guinea, Guinea‐Bissau, Mali, Nigeria, Senegal and Togo.

**Figure 1:**
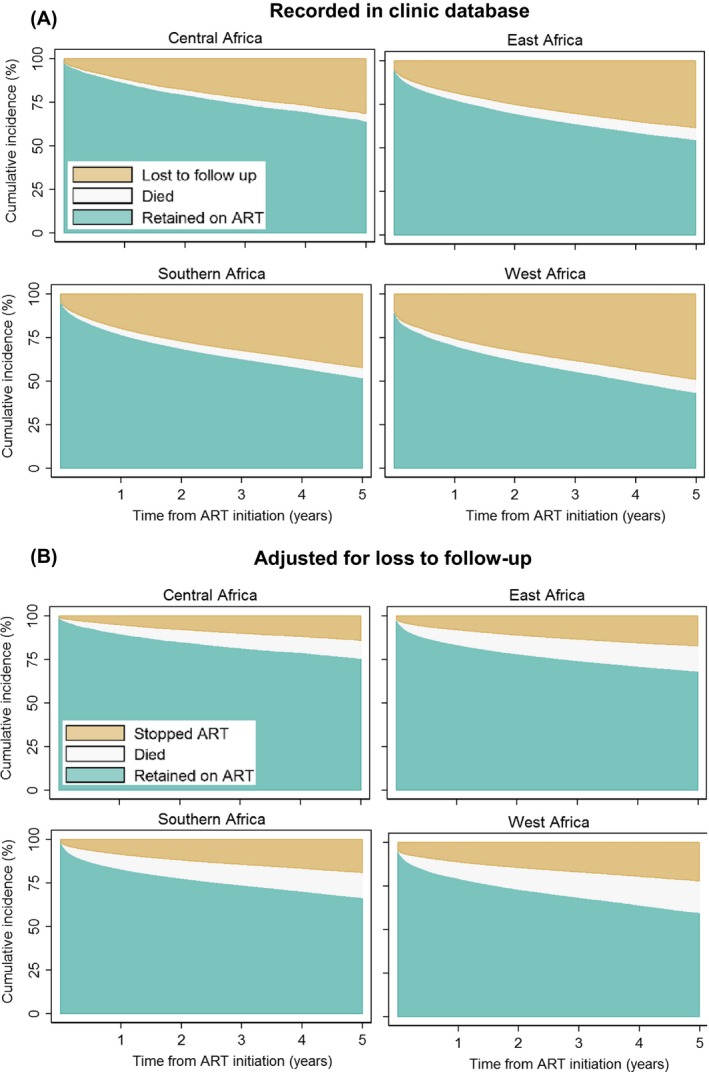
Cumulative incidence of antiretroviral therapy outcomes. Panel A: outcomes recorded in clinic databases. Panel B: outcomes adjusted for unrecorded deaths and transfers among patients lost to follow‐up. Analyses based on 505,634 patients starting antiretroviral combination therapy in Central Africa, East Africa, Southern Africa and West Africa.

Figure 1 shows cumulative retention, loss to follow‐up, and mortality based on the data recorded in the clinics' databases (panel A), and cumulative retention, stopping ART, and mortality from the adjusted analysis that take unobserved deaths and transfers among patients lost to follow‐up into account (panel B). As expected, retention and mortality were higher in the adjusted analysis. Overall, cumulative retention declined from 76.8% (95% CI 76.7 to 77.0) at year 1 to 52.1% (51.9 to 52.3) at year 5 in the crude analysis, and from 83.1% (83.0 to 83.2) to 66.6% (66.4 to 68.8) in the adjusted analysis. Cumulative mortality increased from 3.5% (3.5 to 3.6) in year 1 to 6.0% (6.0 to 6.1) in year 5 in the crude analysis and from 8.4% (8.3 to 8.4) to 14.7% (14.5 to 14.8) in the adjusted analysis. In the adjusted analysis, the percentage of patients who stopped ART increased from 8.5% (8.5 to 8.6) at year 1 to 18.8% (18.6 to 18.9) at year 5. In crude analysis, loss to follow‐up was highest in West Africa and lowest in Central Africa and recorded mortality was below 10% at 5 years across all regions.

In sensitivity analyses adjustment was based on the lower and upper limits of the 95% CIs of the adjustment parameters from the tracing studies 11. Table 2 compares estimates at years 1 to 5 for retention on ART, stopping ART, and mortality from sensitivity analyses with estimates from crude and adjusted analyses. For example, the proportion of people who stopped ART at 5 years was 30.6% based on the lower limit and 1.8% based on the upper limit of the 95% CIs of the adjustment parameters.

**Table 2:** Cumulative incidence of antiretroviral therapy outcomes

	Cumulative incidence of antiretroviral therapy outcomes (95% CI)			
	Recorded in clinic databases^a^	Adjusted with point estimate^b^	Adjusted with lower limits of CI^b^	Adjusted with upper limits of CI^b^
1 year				
Retained on ART	76.8 (76.7 to 77.0)	83.1 (83.0 to 83.2)	79.7 (79.6 to 79.8)	87.5 (87.4 to 87.6)
Lost to follow‐up/stopped ART^c^	19.6 (19.5 to 19.7)	8.5 (8.5 to 8.6)	14.2 (14.1 to 14.2)	0.8 (0.8 to 0.8)
Died	3.5 (3.5 to 3.6)	8.4 (8.3 to 8.4)	6.2 (6.1 to 6.2)	11.7 (11.6 to 11.8)
2 years				
Retained on ART	68.8 (68.7 to 69.0)	77.3 (77.6 to 77.8)	72.9 (72.8 to 73.0)	84.1 (83.9 to 84.2)
Lost to follow‐up/stopped ART^c^	26.7 (26.6 to 26.9)	11.7 (11.6 to 11.8)	19.3 (19.2 to 19.5)	1.1 (1.1 to 1.1)
Died	4.4 (4.4 to 4.5)	10.6 (10.5 to 10.7)	7.8 (7.7 to 7.8)	14.9 (14.8 to 15.0)
3 years				
Retained on ART	62.8 (62.7 to 63.0)	73.8 (73.7 to 73.9)	67.9 (67.7 to 68.0)	81.6 (81.5 to 81.8)
Lost to follow‐up/stopped ART^c^	32.1 (32.0 to 32.3)	14.2 (14.1 to 14.3)	23.3 (23.2 to 23.4)	1.3 (1.3 to 1.4)
Died	5.0 (5.0 to 5.1)	12.1 (12.0 to 12.2)	8.8 (8.7 to 8.9)	17.0 (16.9 to 17.2)
4 years				
Retained on ART	57.5 (57.4 to 57.7)	70.2 (70.1 to 70.3)	63.3 (63.2 to 63.5)	79.5 (79.3 to 79.6)
Lost to follow‐up/stopped ART^c^	36.9 (36.8 to 37.1)	16.4 (16.3 to 16.5)	26.9 (26.8 to 27.0)	1.5 (1.5 to 1.6)
Died	5.6 (5.5 to 5.6)	13.4 (13.3 to 13.5)	9.8 (9.7 to 9.9)	19.0 (18.8 to 19.1)
5 years				
Retained on ART	52.1 (51.9 to 52.3)	66.6 (66.4 to 68.8)	58.7 (58.5 to 58.9)	77.4 (77.2 to 77.5)
Lost to follow‐up/stopped ART^c^	41.8 (41.6 to 42.0)	18.8 (18.6 to 18.9)	30.6 (30.4 to 30.8)	1.8 (1.7 to 1.8)
Died	6.0 (6.0 to 6.1)	14.7 (14.5 to 14.8)	10.6 (10.5 to 10.7)	20.8 (20.7 to 21.0)

Data are cumulative incidences of antiretroviral therapy outcomes (in %) and 95% confidence intervals for patients starting antiretroviral therapy. Time is measured in years from start of antiretroviral therapy.

a Crude estimates show cumulative incidence of death, loss to follow‐up and retention on ART as recorded in the clinic database.

b Adjusted estimates correct for underreporting of mortality and transfer out based on the point estimates and 95% confidence intervals (CIs) for mortality (20.8%, 95% CI: 11.3 to 35.1%) and self‐transfer (35.9%, 95% CI: 16.8 to 60.9%) among patients lost to follow‐up. Adjustment parameters are derived from a meta‐analysis of tracing studies 11.

c In the adjusted analyses patients alive but not retained on ART are assumed to have stopped ART.

We analysed data from over 500,000 HIV‐infected children and adults who initiated ART in 22 countries in sub‐Saharan Africa. We adjusted for unrecorded deaths and silent transfers among persons lost to follow‐up and found that retention on ART was considerably higher in the adjusted analysis. We found that an estimated 19% of people who initiated ART had stopped ART by 5 years. Cumulative mortality was 15% at 5 years, substantially higher in the adjusted analysis than the analysis based on recorded deaths. The sensitivity analyses showed that estimates for ART outcomes were sensitive to the choice of adjustment parameters.

Our study has important implications for monitoring outcomes of the scale‐up of ART in sub‐Saharan Africa in general, and particularly monitoring progress towards the 90‐90‐90 targets. The second of the 90‐90‐90 targets states that at least 90% of people diagnosed with HIV should be receiving ART. Our study could not assess this target directly because no data on the number of people diagnosed with HIV during the study period were available. However, both patients stopping and patients not starting ART will compromise the second 90 target. Our study shows that after several years of ART close to 20% of all patients starting ART have stopped treatment. If WHO guidelines for universal immediate ART are successfully implemented 3, retention on ART will be one of the key determinants of whether or not the second 90 target is met. Of note, a recent review of national HIV care continua found that only one country in sub‐Saharan Africa, Swaziland, met the second of the three 90 targets, and two other countries, Rwanda, and Namibia, were close to achieving the target 19.

Our analysis has several limitations. We implemented WHO's definition of loss to follow‐up and only consider the last recorded status of patients at closure of database. Patients with transient treatment interruptions who returned to care were considered as retained throughout follow‐up. Therefore, our analysis does not take into account transient treatment interruptions and retention estimates reflect the final status of patients at database closure 20. Our analysis therefore may overestimate retention. Transient treatment interruption could be taken into account as intermediate states in multistate models, but heterogeneity of data makes it difficult to implement such analysis in multi‐cohort studies 21, 22. Overall, 40% of people living with HIV were classified as lost to follow‐up in the clinical databases of the ART programmes. These patients may have discontinued ART, self‐transferred to another programme, or died but their true outcomes are unknown. Unrecorded deaths can be ascertained and mortality estimates corrected by tracing patients lost to follow‐up or by linking the clinical databases to the vital registry 23, 24. We used inverse probability weighting to adjust for undocumented mortality and self‐transfers, based on the best available data from a meta‐analysis of tracing or linkage studies performed in sub‐Saharan Africa 11. We assumed that the pooled estimates from the meta‐analysis were applicable to the IeDEA data in all regions. The meta‐analysis included tracing studies from several countries in the East and Southern African IeDEA regions, but only a few tracing studies from the West and Central African IeDEA regions were available. We are thus more confident about the generalizability of the adjustment parameters to East and Southern African IeDEA data, than for the West and Central Africa regions. The goal of our study was to describe the likely underestimate of mortality and retention in care at the regional level. Our adjustment did not account for facility‐ or individual‐level characteristics, although ART outcomes may vary by setting, clinic, and according to individual‐level characteristics such as CD4 cell count, age, gender, or pregnancy 4, 7, 15, 25, 26, 27. Future studies should try to account for these factors. We have begun tracing random samples of all patients lost to follow‐up using a standardized protocol in IeDEA Southern Africa to generate adjustment factors for specific clinics in IeDEA. However, the pooled estimates from the meta‐analysis are currently the best available data to adjust our estimates. A further limitation of our analysis is that the confidence intervals of the adjusted estimates did not account for the statistical uncertainty around adjustment parameters. However, we showed how uncertainty around these parameters affected estimates for ART outcomes in sensitivity analyses. Our study included some rural primary care clinics, but larger urban secondary or tertiary care hospitals dominated and our data are unlikely to be representative of all ART programmes in the countries involved 17. A further limitation of our adjustment parameters is that data on self‐transfer from tracing studies is usually self‐reported and not verified by clinical records. Over‐reporting of self‐transfer may lead to overestimation of retention in care.

## 4 Conclusion

Efforts to retain people living with HIV in long‐term care and to bring patients back to care must be intensified, both to improve outcomes in individuals and to prevent HIV transmissions at the population level 5. Monitoring of progress must take unrecorded mortality and silent transfers between clinics into account.

## Competing interests

The authors declare that they have no conflicts of interest to disclose.

## Authors' contributions

EZ, AH, and ME wrote the first draft of the study protocol; all authors contributed to the final version of the protocol. AH, EZ, and NA did statistical analyses, with interpretation of results by ME, AH, EZ, and NF. AH, and EZ wrote the first draft of the report, which was revised by ME, NF, MF, MV, MD, AE, and AA; all authors commented on earlier drafts of the report. IeDEA data centres. FD, DN, JS, TN, AT, and AP assisted in implementation, fieldwork, and data collection at study sites and regional data centres. ME obtained funding for the project. All authors reviewed and approved the final version for submission.

## Funding

The African regions of the International epidemiology Databases to Evaluate AIDS (IeDEA) are supported by the National Cancer Institute (NCI), the Eunice Kennedy Shriver National Institute of Child Health and Human Development, the National Institute of Allergy and Infectious Diseases (NIAID), the National Institute of Mental Health (NIMH), and the National Institute on Drug Abuse (NIDA) as part of IeDEA (grants 5U01AI069919‐04, 5U01‐AI069924‐05, 1U01 AI069927, and U01AI069911‐01). This study was also supported by the Bill and Melinda Gates Foundation through the Measurement and Surveillance of HIV epidemics (MESH) consortium. MPF was funded through Cooperative Agreement AID 674‐A‐12‐00029 from the United States Agency for International Development.
